# A Histone Map of Human Chromosome 20q13.12

**DOI:** 10.1371/journal.pone.0004479

**Published:** 2009-02-20

**Authors:** Pelin Akan, Martin Sahlén, Panagiotis Deloukas

**Affiliations:** 1 Wellcome Trust Sanger Institute, Wellcome Trust Genome Campus, Hinxton, Cambridge, United Kingdom; 2 Astronomy Centre, University of Sussex, Brighton, United Kingdom; University of Munich and Center of Integrated Protein Science, Germany

## Abstract

**Background:**

We present a systematic search for regulatory elements in a 3.5 Mb region on human chromosome 20q13.12, a region associated with a number of medical conditions such as type II diabetes and obesity.

**Methodology/Principal Findings:**

We profiled six histone modifications alongside RNA polymerase II (PolII) and CTCF in two cell lines, HeLa S3 and NTERA-2 clone D1 (NT2/D1), by chromatin immunoprecipitation using an in-house spotted DNA array, constructed with 1.8 kb overlapping plasmid clones. In both cells, more than 90% of transcription start sites (TSSs) of expressed genes showed enrichments with PolII, di-methylated lysine 4 of histone H3 (H3K4me2), tri-methylated lysine 4 of histone H3 (H3K4me3) or acetylated H3 (H3Ac), whereas mono-methylated lysine 4 of histone H3 (H3K4me1) signals did not correlate with expression. No TSSs were enriched with tri-methylated lysine 27 of histone H3 (H3K27me3) in HeLa S3, while eight TSSs (4 expressed) showed enrichments in NT2/D1. We have also located several CTCF binding sites that are potential insulator elements.

**Conclusions/Significance:**

In summary, we annotated a number of putative regulatory elements in 20q13.12 and went on to verify experimentally a subset of them using dual luciferase reporter assays. Correlating this data to sequence variation can aid identification of disease causing variants.

## Introduction

Gene regulation is a complex process, requiring a large number of proteins acting cooperatively on regulatory DNA sequences which in turn are wrapped by histones. While instructions for the recruitment of specific factors onto regulatory sites are partially stored in the sequence, DNA should also be decorated with the correct epigenetic markers to permit functional interactions between trans-acting factors and DNA. Chromatin immunoprecipitation (ChIP) is a powerful technique which takes a snapshot of the genome together with its associated proteins. This method is currently the common method of choice (i) to map proteins onto their genomic targets [1]–[5] and (ii) to understand the chromatin context of functional genomic sites [6]–[12]. Histone tails can be covalently modified by acetylation, phosphorylation, sumoylation, ubiquitination and methylation. It is proposed that certain combinations of histone modifications, so called the histone code, partially sets the appropriate environment for cellular machineries to act upon their target DNA sequences [13]. It was shown that in humans 5′ ends of transcriptionally active genes are enriched with H3K4me3 and acetylated H3 [7], [12], [14]–[17], while transcriptionally repressed regions carry modifications such as tri-methylated lysine 9 (H3K9me3) and 27 of histone H3 [10], [12], [18]–[21]. H3K4me2 has been found on promoter of genes that are poised for expression in undifferentiated and pluripotent cells, serving as a preparatory signal for the start of transcription for lineage commitment [22], [23].

In embryonic stem cells, key developmental genes are occupied by nucleosomes trimethylated at H3K27 and this modification is required for silencing of such genes by polycomb repressor complexes (PRCs) [18], [19]. PRCs bind to developmentally regulated genes and ensure their correct expression patterns to preserve the pluripotency of cells. Although this silencing mechanism is not yet clear, it seems that H3K27me3 facilitates binding of such complexes in Drosophila [24]. Another study showed that decreasing levels of H3K27me3 on promoter regions of certain silenced genes is associated with their activation which may contribute to oncogenesis depending on the functional nature of erroneously activated silenced genes [25]. X-chromosome inactivation in Drosophila females has also been linked to tri-methylation of H3 at lysine 27 [11].

Promoters of key developmental genes often carry both activatory (H3K4me3) and repressive (H3K27me3) histone marks in embryonal stem cells [26]. Such regions are now called bivalent domains, and they appear to be important to preserve the differentiation potential of stem cells by keeping key developmental genes silent but poised for activation upon initiation of specific differentiation routes. Indeed this bivalent marking of such regions is lost in differentiated cells where they either have repressive or active marking [27].

Mono-methylation at lysine 4 (H3K4me1) is often associated with active chromatin in eukaryotes and target regions for binding of chromodomains involved in chromatin structure [28], [29]. This modification is also found on distal regulatory elements [12], [30].

CTCF is a versatile protein functioning as an activator or repressor on promoters or silencer sequences, or a chromatin insulator protein [31]. It is also located as part of multi-protein complexes regulating histone acetylation and deacetylation [32].

Human chromosome 20q13.12, (chr20:41,600,001–45,800,000, NCBI build 36) is a gene rich region that has been associated with a number of medical conditions such as diabetes [33]; obesity [34]–[36]. It harbours a commonly deleted region (CDR) found in patients with myeloproliferative disorders and myelodisplastic syndromes [37], and ovarian cancer [38].

In this study, a 3.5 Mb region on 20q13.12 was investigated using ChIP experiments performed with eight different antibodies (Table 1) in two cell lines: HeLa S3, a cervical carcinoma, and NT2/D1, a pluripotent embryonal carcinoma cell line. We profiled three methylation status of H3K4 to map novel and known promoters as well as distal regulatory elements. RNA polymerase II antibody was used to investigate actively transcribed regions. Antibody against H3K27me3 was used to map transcriptionally silenced and/or developmentally regulated genes. Acetylated histone H3 and H4 antibodies were also included in the study to further understand their correlation with histone methylation. Lastly, we have used CTCF antibody to map potential insulator elements. The enrichment profile of each antibody was mapped using a custom-made tiling microarray with a resolution of 1.8 kb (2067±483 bp) (ArrayExpress Accession Number A-MEXP-1441) and correlated to a detailed transcript map already available for this region.

**Table 1:** The list of antibodies used in ChIP experiments.

Antibody	Abbreviation	Expected Genomic Regions
CTD of RNA polymerase II (phosphorylated at Serine 5)	PolII	Actively transcribed regions
mono-methylated Histone H3 at lysine 4	H3K4me1	Distal regulatory regions
di-methylated Histone H3 at lysine 4	H3K4me2	active promoters
tri-methylated Histone H3 at lysine 4	H3K4me3	active promoters
tri-methylated Histone H3 at lysine 27	H3K27me3	Silenced genes and heterochromatin?
acetylated Histone H3 at lysine 9 and 14	H3Ac	active promoters
acetylated Histone H4 at lysine 5,8,12 and 16	H4Ac	Chromatin remodeling
CTCF	CTCF	mainly insulator elements

## Materials and Methods

### Cell Lines

HeLa S3, human cervical carcinoma cell line, was kindly provided by Cancer Genome Project at the Sanger Institute. NTERA-2 clone D1 (NT2/D1), human Caucasian pluripotent embryonal carcinoma cell line purchased from European Collection of Cell Cultures (ECAC). Both cell lines were grown in high glucose (5 g/l) DMEM (Gibco, #41966029) supplemented by 10% FBS and 1× antibiotic/antimycotic solution (Gibco, #10378016) in a humidified incubator at 37°C and 5% CO_2_.

### Chromatin Immunoprecipitation

ChIP experiments for all cells were carried out as described in Koch (2007) [39]. Briefly, ∼5×10^7^ cells were fixed with 0.37% or 1% formaldehyde. For histone modified antibodies, chromatin is crosslinked for 15 minutes with 0.37% formaldehyde (FA). For PolII antibody, chromatin is crosslinked for 1 hour with 1% FA and for CTCF the chromatin is crosslinked for 15 minutes with 1% FA. Cells were lysed, collected nuclei was lysed with nuclear lysis buffer. Chromatin was fragmented with a Sanyo Soniprep sonicator to a size range of 300–700 bases with 30 seconds bursts (Figure S1). After removal of a control aliquot (input chromatin), diluted chromatin was incubated at 4°C overnight with relevant antibodies (CTCF (sc15914, Santa Cruz Biotechnology), H3K4me1 (ab8895, Abcam), H3K4me2 (ab7766, Abcam), H3K4me3 (ab8580, Abcam), H3K27me3 (ab7312, Abcam), H3Ac (at K9 and 14) (06-599, Upstate Biotech), H4Ac (at K5,8,12 and 16) (06866, Upstate Biotech), PolII (ab513, Abcam), Rabbit IgG (12-370, Upstate Biotech), Goat IgG (Santa Cruz Biotechnology, #sc-3850)). Immune complexes were then precipitated with Agarose Protein A/G beads, washed and eluted. Crosslinking of antibody/chromatin and input chromatin samples were reversed by incubation at 65°C for 6 hours. Samples were treated with Proteinase K overnight at 45°C. DNA was recovered with water saturated phenol and chloroform, then ethanol-precipitated.

### Construction of Tilepath Arrays

1800 overlapping plasmid clones (2 kb inserts) were selected from a sequenced chromosome 20 library in pUC18 [40]. Inserts were amplified with AmpliTaq Gold Polymerase and pUC18 primers (95°C for 5 min then 30 cycles of 1 min at 95°C, 1 min at 60°C, and 4 min 72°C); forward primer contained an amino group linked to sixth carbon atom at the 5′ terminal for attachment onto microarrays. Each fragment was printed in triplicate on the array. The array and experiment design can be accessed on ArrayExpress (http://www.ebi.ac.uk/microarray-as/aer/login) using the accession number A-MEXP-1441.

### Quantitative PCR

We have validated the enrichment signals of 37 regions using quantitative PCR for all antibodies. Quantitative PCR was performed according to manufacturer's instructions (Eurogentec, RT-SN2X-03+WOUN) on an ABI PRISM® 7700 – PCR: 2 mins at 50°C followed by 10 min at 95°C, then 40 cycles of 15 sec at 95°C and 1 min at 60°C. Amplification levels of diluted input chromatin were used as the baseline to determine amplification levels of the ChIP samples. Ct values and amplification curves were generated by ABI PRISM® 7700 analysis software. Primer sequences are available upon request.

### Labelling, Hybridisations and Array Scanning

ChIP DNA (ChDNA) and input chromatin (IP) were labelled with cyanine-3 (Cy3) and cyanine-5 (Cy5) respectively by random priming (BioPrime Labelling Kit, Invitrogen, #18094-011). Unlabelled nucleotides were removed using micro-spin G50 columns (Pharmacia Amersham #275330-01). Equal amounts of Cy3-IP and Cy5-ChDNA were mixed with human Cot1 DNA to suppress repeat regions and precipitated with ethanol. Array hybridisation was for 45 h at 37°C in the dark on a Tecan HS4800 Pro® station. Slides were washed with 1×PBS/0.05% Tween20 and 0.1× SSC and HPLC purified water, dried on the station and scanned using ScanArrayExpress HT (Perkin Elmer). Light intensities on each spot on the array were digitalised with ProScanArray® Express software (Perkin Elmer) and ratio of Cy5 (experiment) to Cy3 signal (IP) is assigned as the signal of the corresponding spot.

### Data Analysis

Three biological and three technical replicates were performed for each antibody. Average signal values were calculated for each antibody (experiment) and its isotype antibody (control). The data was floored and centred by dividing each data point by the median of the dataset and log2-transformed. When the normalised signal of a spot is 2.5 times higher than the standard deviation of the data, it is considered enriched. Quantitative PCR of 37 regions confirmed enrichment over input chromatin for 100% of the peaks assigned using the above enrichment cut-offs for all antibodies.

### Affymetrix Expression Arrays

GeneChip™ Human Genome U133A 2.0 Expression Analysis Arrays (Affymetrix, #900466) were used to determine the gene expression profile of HeLa S3 and NT2/D1 cell lines; expression values were calculated by GeneSpring GX Software (Agilent).

### Dual Luciferase Assays

Candidate promoters were cloned into pGL3-basic (#E1751) and pGL3-enhancer (#E1771) vectors. pGL3-promoter (#E1761) and pGL3-control vector (#E1741) were used as positive controls. pRL-SV40 vector (#E2231) was used as the internal control. We also tested activity of inter-genic DNA fragments (of similar size) with no known function to determine the background (negative controls). Promoter constructs were transfected to HeLa S3 and NT2/D1 cells using QIAGEN Effectene Transfection Reagent (QIAGEN, #301425) according to manufacturer's instructions. A dual luciferase assay (Promega, #E1910) was used to measure the luciferase and renilla activity within the transfected cells. Two days after transfection, growth media was removed and cells were washed with 100 µl of 1×PBS. Then, cells were incubated in 1× Passive Cell Lysis Buffer for 20 min at room temperature for cell lysis. Luciferase assays were performed with MicroLumat Plus LB 96V luminometer (Berthold Technologies). First, 50 µl of Luciferase Reagent II was injected to the lysate and luciferase activity was measured for 10 sec. Then, 50 µl of *Stop and Glo*® reagent was injected to the lysate to stop the luciferase activity and catalyse the renilla reaction, incubated for 1.6 sec and then renilla activity was measured for 10 sec. All experiments were performed in triplicates and repeated twice.

## Results

**Figure 1:**
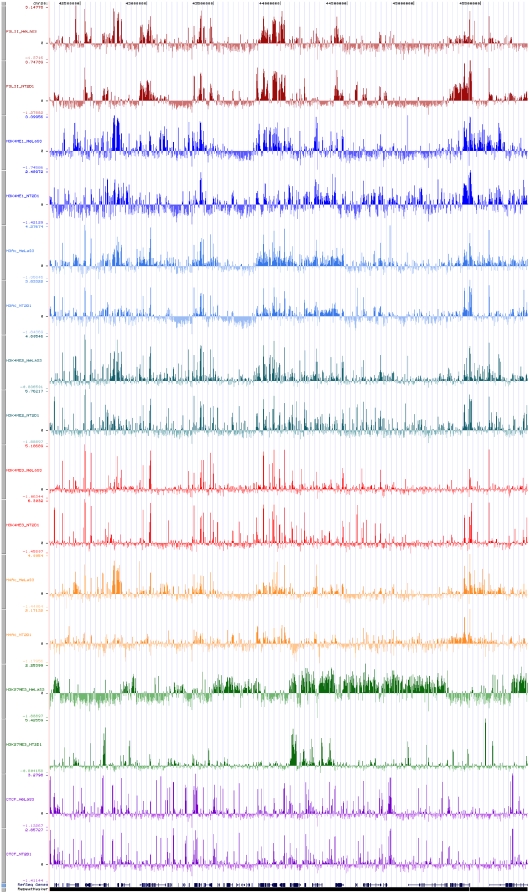
Normalised signals of all antibodies in both cell lines displayed in UCSC Genome Browser as custom tracks across human 20q12–13.2.

In order to identify regulatory elements across a 3.5 Mb region of human chromosome 20q13.12, we profiled two cell lines by ChIP experiments against RNA polymerase II, CTCF and six histone modifications (Table 1). Enrichment was measured with a 2 kb tilepath array spanning the region (see Methods). The enrichment pattern of the region in both cell lines is shown in Figure 1. The histograms of the enrichment signals of all antibodies in both cell lines are shown in Figure S2. We also surveyed gene expression levels of the 72 protein-coding genes in the region using Affymetrix expression arrays and found 28 and 35 genes to be expressed in the HeLa S3 and NT2/D1 cell line respectively.

### Enrichment Profile of the TSS regions

In HeLa S3 cells, the regions containing the transcription start sites (TSS)s of 11 genes showed enrichment with PolII antibody. In addition, regions containing the TSSs of 10 genes showed enrichment only with H3K4me3 (Table S1). PolII and/or H3K4me3-enriched genes were highly or moderately expressed although there were four lowly expressed genes that also showed enrichments with either with PolII or H3K4me3.

In NT2/D1 cells, 28 genes showed enrichment with either PolII or H3K4me3 with 13 having both (Table S2). All enriched genes except two (SLC35C2 and C20ORF165) were highly or moderately expressed.

Of the regions which contained a TSS and were enriched with PolII and/or H3K4me3, nearly all showed enrichment with H3Ac in both cell lines (Table S1 and S2). H4Ac enrichment was observed in regions containing active TSSs only half the time (both cell lines) which is expected since it is mainly associated with chromatin structure changes and protein interactions [41].

**Figure 2:**
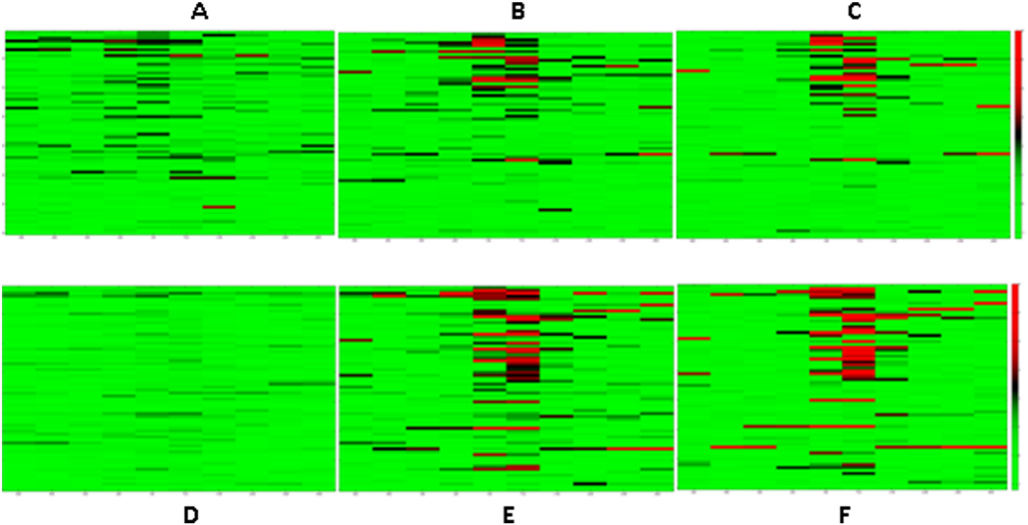
Heat maps of H3K4me3 (A, D), H3K4me2 (B, E) and H3K4me1 (C, F) signals of 70 transcripts (sorted according to their gene expression levels, the names of the genes can be found in the supplementary table 1 in the same order as here) in HeLa S3 cells and NT2/D1 cells respectively.

**Figure 3:**
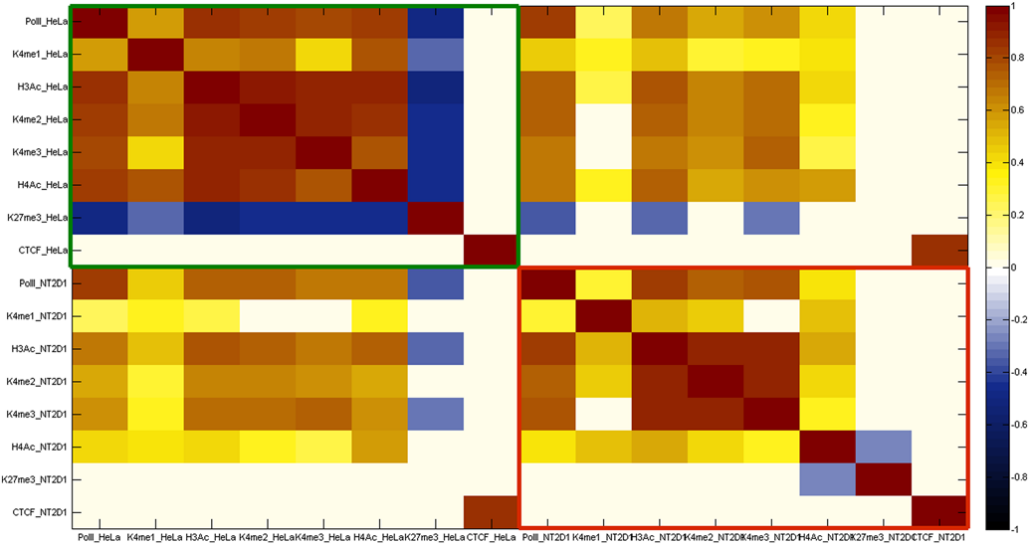
Pairwise correlation coefficient matrix of enrichment signals for all antibodies on the spots containing TSSs in HeLa S3 cells (green rectangle), NT2/D1 cells (red rectangle), and between the two cell lines (remaining areas). Note that only coefficients which are statistically significant at the 95% level, according to a standard *p*-test are shown as non-zero. The full correlation matrix without this significance cut is provided in Figure S3.

Figure 2 shows the mono-, di- and tri-methylation profiles of histone H3K4 within 10 kb distance of the start site of all the genes. H3K4me1 does not appear to have a location preference; the enrichment levels do not change greatly around the TSS especially in NT2/D1 cells. Exceptions include *PRKCBP1*, which is enriched with H3K4me1 up to 6 kb either direction of its start site as well as *NCOA3*, *ZNF335* and *SDC4*, which are enriched across downstream sequences. Our results suggest that active promoter regions are clearly marked with either H3K4me3 or H3K4me2 whereas H3K4me1 does not have the same discriminatory power for active TSSs. We have also investigated the pairwise correlation of raw enrichment signals of each antibody in each cell line and between the cell lines. Figure 3 displays pairwise linear correlation coefficients for each pair of antibody signals on the spots containing TSSs. Note that only those coefficients that are significant at the 95% level based on a standard p-test are shown as non-zero. The H3Ac, H3K4me2 and H3K4me3 enrichment levels were correlated well with each other in both cells but not with H3K4me1. Notably, H3K4me1 signals were positively correlated with H3K4me3 signals in HeLa S3 cells, but there was no significant correlation found between H3K4me1 and H3K4me3 in NT2/D1 cells. The H3K27me3 signals were negatively correlated with those of H3K4me2, H3K4me3 and H3Ac only in HeLa S3 cells, whereas there was no or little positive correlation in NT2/D1 cells. This observation suggests a differential usage of H3K27me3 modification between the two cell lines. We have also included the correlation matrix of all signals in both cell lines in Figure S4.

**Figure 4:**
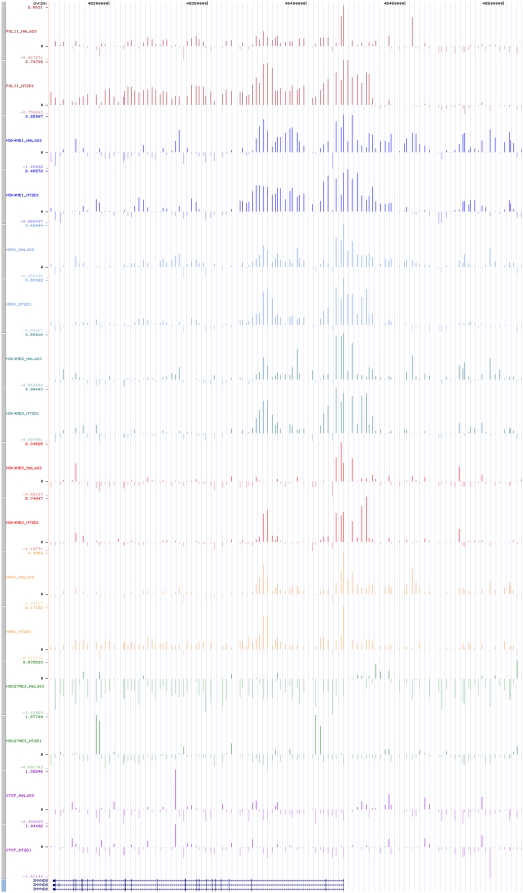
Enrichment pattern of PRKCBP1 (ZMNYD8) gene and its upstream region (chr20:45,270,000–45,510,000) with all antibodies in both cell lines.

The above analysis identified novel promoters. A region 10 kb upstream of *PRKCBP1* (chr20:45,430,683–45,431,820 bp) showed both strong PolII and H3K4me3 enrichment in NT2/D1 cells (Figure 4). In VEGA Genome Browser (http://vega.sanger.ac.uk), this region harbours the TSS of a GENSCAN prediction supported by the 5′ end of a human spliced EST (CN335160) from embryonic stem cells. To test whether this region is a tissue-specific alternative *PRKCBP1* promoter, we cloned 1137 bp in a luciferase reporter vector and transfected NT2/D1 cells. Figure S5 shows that this fragment has indeed promoter activity.

In the same cell line we also found a strong H3K4me3 enrichment in the second intron of *SULF2* (chr20:45,818,232–45,819,183 bp). Here, ENSEMBL annotation revealed the presence of a promoter prediction (FirstExon) and a mouse EST [42]. Constructs of this fragment were assessed for promoter activity in both cell lines (Figure S6) and the results further supported its function as an NT2/D1-specific alternative *SULF2* TSS.

### Partial histone code of distal regulatory elements

**Figure 5:**
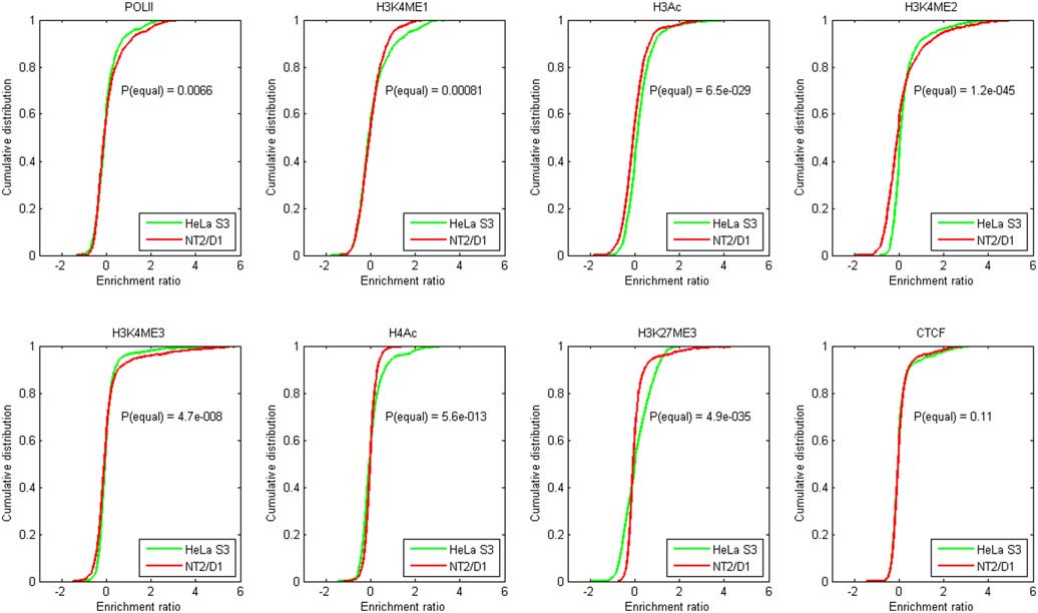
Empirical cumulative distribution functions for all antibodies, for the two different cell lines (HeLa S3 and NT2/D1), using the full data set. The probability of the two cell-line distributions being equal, according to a two-tailed Kolmogorov-Smirnov test, is shown in each panel (the null hypothesis being that the distributions are equal, and the alternative hypothesis that they are not equal).

We assessed five histone modifications, namely H3K4me1, H3K4me2, H3K4me3, H3Ac and H4Ac and found them to display strikingly different profiles between the two cell lines. Figure 5 shows the empirical cumulative distribution functions (i.e. the integrated enrichment signal distributions) for all antibodies in the two cell lines. Based on these, we have used a two-tailed Kolmogorov-Smirnov test to find whether the distributions for a particular antibody are equal in the two cell lines. This test is very general and does not assume any particular distribution of data, e.g. normal distribution. The null hypothesis that the distributions are equal is tested against the hypothesis that they are not equal. The resulting asymptotic probabilities that the distributions are equal are shown in Figure 5. In all cases, except CTCF, the probability that the distributions are different is greater than 99.9%.

**Figure 6:**
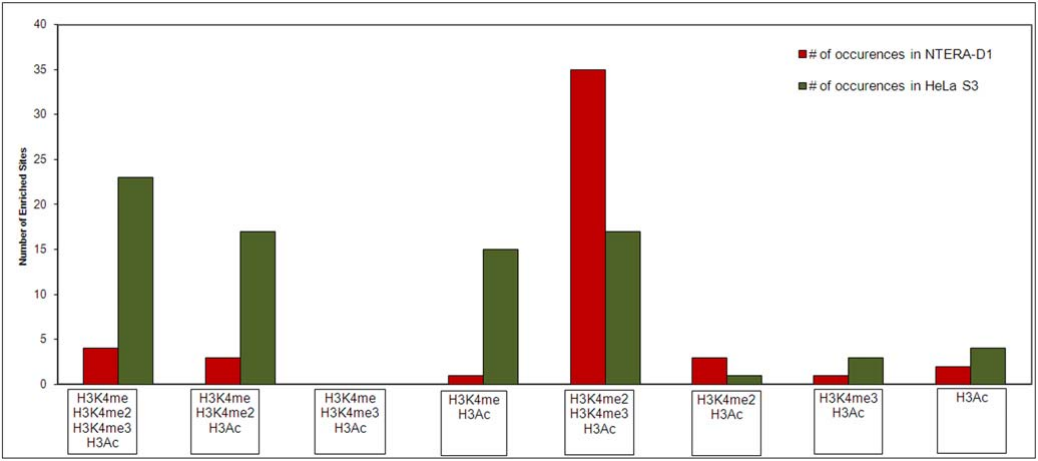
Plots of number of occurrences of possible modified histone combinations with H3Ac in HeLa S3 and NT2/D1 cells respectively.

Across the region, no site showed enrichment with H3K4me1+H3K4me3 or H3K4me1+H3K4me3+H3Ac, most likely due to the fact that H3K4me2 is the intermediate step between H3K4me1 and H3K4me3 [43]. HeLa S3 had three fold more H3K4me1-enriched sites than NT2/D1 cells (Figure 6). Among the sites that were only enriched with H3K4me1, 80% (in HeLa S3) and 85% (in NT2/D1) were located within inter- or intra-genic regions. Ten out of 11 HeLa S3 sites (90%) and 40 out of 54 NT2/D1 (73%) H3K4me2-enriched sites were located within inter- or intra-genic regions indicating that they do not possess the potential for further methylation. This observation suggests that H3K4me2 may also play a functional role for the regulation of distal regulatory or structural elements other than TSSs.

Sites that were enriched with H3K4me1+H3K4me2 were not close to annotated TSSs, while 90% of the sites that were enriched with H3K4me1+H3K4me2+H3Ac were close or overlapping TSSs in both cells. This observation can lead to two conclusions; (i) H3 acetylation may indeed have a stimulatory effect for a site to be enriched in H3K4me3 [44], or (ii) H3Ac may not be a common histone modification in regulatory elements other than promoters.

**Figure 7:**
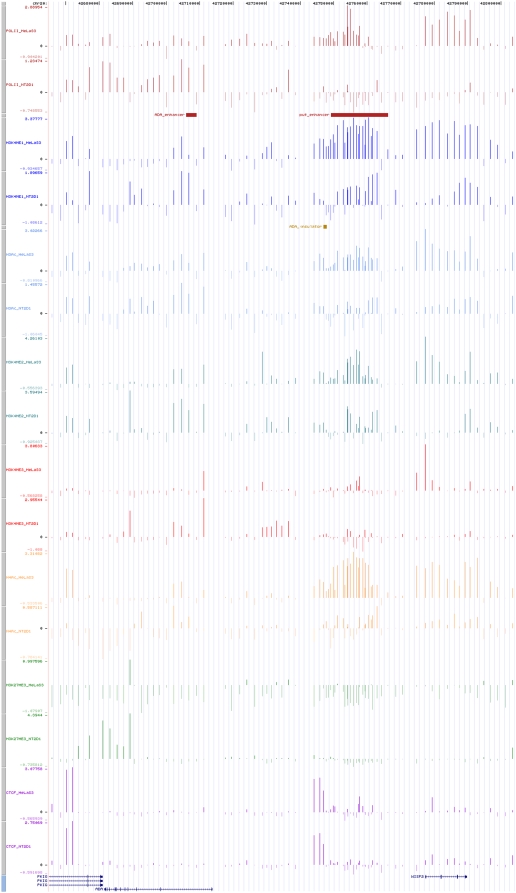
Enrichment profile of the 140 kb region (chr20:42,665,000–42,805,000) containing one known and one putative enhancer and one putative insulator with all antibodies in both cell lines.

One region lying within the first intron of the *ADA* (42,704,657 to 42,706,871 bp) showed enrichment with H3K4me1 and H3K4me2 in HeLa S3 cells (Figure 7). The same region and its 2 kb upstream and downstream flanks (42,702,249 to 42,708,995) also showed H3K4me1 and H3Kme2 enrichment in NT2/D1 cells. A known intronic enhancer of the *ADA* promoter (42,706,026 and 42,709,030 bp) is located in this region containing three DNase hypersensitive sites [45]. This observation supports the hypothesis that mono-methylated histone H3 could be a marker for distal enhancer elements [12], [30].

A high number of sites containing or overlapping conserved non-genic sequences (CNGs) were found to be enriched with H3K4me1, H3K4me2, H3Ac or H4Ac in both cell lines. The region, chr20:42,749,148–42,766,245 bp, which is annotated to only harbour a non-coding novel transcript (RP11-445H22.4), displayed a complex enrichment profile only in HeLa S3 cells (Figure 7). The first two kilobases of this region is enriched with PolII but there is no H3K4me3 enrichment suggestive of a tissue specific distal regulatory element whose function is regulated by histone modifications. The PolII enrichment may reflect an interaction of this region with the initiation complex on the promoter of its target gene located elsewhere in the genome which could lead to cross-linking and co-immunoprecipitation with the initiation complex (i.e. PolII acts as a bridge between the two sequences). Note that this region was only enriched with H3K4me1 and H3K4me2 in NT2/D1 suggesting that this hypothetical element could be inactive in this cell line. To investigate its putative enhancer activity, the region was cloned to an enhancer-testing vector and transfected to both cell lines. We found moderate enhancer activity in both transfected cell lines which supports our hypothesis of an element with distal regulatory activity (Figure S7). However, the fact that it shows moderate enhancer activity in both cell lines suggests that tissue specificity is most likely regulated by chromatin modifications and further experiments are required to test this hypothesis.

### H3K27me3 enrichment profile varies greatly between the cell lines

ChIP-chip experiments carried out in HeLa S3 cells revealed 17 regions enriched with H3K27me3 of which none was close to an annotated TSS (Table S4). In NT2/D1 cells, we found enrichment in 75 inter- and intra-genic regions as well as in the TSSs of eight genes (4 expressed); three also have H3K4me3 enrichment (Table S5). Except one intra-genic region, none of the H3K27me3-enriched regions showed enrichment with PolII or H3Ac or H4Ac (both cell lines). Spatially, H3K27me3-enriched regions in HeLa S3 confined within circa 1 Mb (right end) whereas they were scattered across the entire region in NT2/D1 (Figure 1).

*SLC12A5*, which encodes for a membrane protein responsible for co-transporting potassium and chloride ions across the cell membrane and its expression is restricted to neurons in the central nervous system and retina playing an important role in neuronal development [46]. We note that the core promoter of *SLC12A5* did not show any activity in dual luciferase promoter assays we performed (both cell lines: data not shown). As expected *SLC12A* was not expressed in either cell line, but it showed very different enrichment patterns between the cell lines (Figure S8). In NT2/D1 cells, TSS-containing and downstream flanking spots were enriched with H3K4me3, H3Ac and H3K27me3 and the gene body is enriched with H3K27me3 across its sequence. In HeLa S3 cells, only the downstream TSS flanking region was enriched with H3K4me3 and H3Ac. The *SLC12A5* promoter can be classified as “bivalent” in NT2/D1 cells since it appears enriched in both H3K27me3 and H3K4me3 [26]. We also found moderate H3K4me3 and strong H3K4me2 enrichment on the spot carrying the 3′ UTR (NT2/D1 cells only). The 3′ UTR of *SLC12A5* lies within a CpG island (length = 1856 bp, GC% = 64.3, Obs(CpG)/Exp(CpG) = 0.757) which overlaps a promoter (FirstExon) and TSS prediction (Eponine). A possible explanation is that this putative promoter drives an antisense RNA, a mechanism to ensure that the gene is inactive [47].

Another membrane protein, *CDH22* (cadherin-like 22) whose expression is restricted to brain and plays a role in neuronal and non-neuronal cell development [48], showed H3K27me3 enrichment both at the TSS and around it together with H3K4me2 and H3K4me3 (NT2/D1 cells only). Therefore, its promoter is also classified as “bivalent”. In HeLa S3 we found PolII enrichment along with several other activation histone modifications in the sixth intron of *CDH22* (chr20:44,269,828–44,269,597), which might be a potential silencer.

All the H3K27me3 enriched genes have tissue or developmental specific expression profiles, and they have the same expression profile in HeLa S3 cells except CDH22, which is expressed in NT2/D1 but not in HeLa S3. The difference in H3K27me3 enrichment profile between the two cell lines might be due to their different origin, as NT2/D1 is established from a malignant germ line tumour and can differentiate to neurons whereas HeLa S3 cells do not have such ability.

### Several inter- and intra-genic sites were enriched with CTCF

CTCF is a versatile transcription factor functioning as an activator or repressor on promoter or silencer sequences, or a chromatin insulator protein (reviewed in [31]). It is also located as part of multi-protein complexes regulating acetylation status of histones on promoters [32].

We obtained CTCF enrichment in several inter- and intra-genic regions in both HeLa S3 and NT2/D1 cells. Interestingly, more than 95% of the CTCF-enriched sites were common and the overall enrichment signal distributions were similar between the two cell lines (Figure 5). The statistical significance of the signal distributions being equal is about 10%, and hence we cannot conclude that they are equal – but equally neither that they are different. In total, 40 inter-genic and 18 intra-genic regions showed enrichment with CTCF in both cell lines.

None of the eight genes, whose start site was enriched with CTCF, was expressed in HeLa S3 cells whereas in NT2/D1, start sites of three expressed and two non-expressed genes were enriched with CTCF (Table S6). Such enrichment pattern suggests a silencing role for CTCF in HeLa S3 cells. The TSS of *ZNF335* which is not expressed in HeLa S3 was enriched with PolII, H3K4me3 and H3Ac. This gene encodes for a zinc finger protein which indirectly activates ligand-bound nuclear hormone receptors [49]. Its core promoter showed activity in luciferase assays only when cloned with the SV40 enhancer (HeLa S3 cells, data not shown). Since we detected no expression of ZNF335 in this cell line despite the fact that the promoter region seems to be actively transcribed, there should be a mechanism which can either halt the initiation complex or decrease the mRNA stability. Importantly, three intronic sites within *ZNF335* were enriched with CTCF in HeLa S3 cells. It is known that CTCF can act as a repressor by having a negative effect on the elongation process of the transcription [50] and the enriched intra-genic sites might be the mediator of such a mechanism regulating ZNF335 expression. In NT2/D1 where this gene is expressed, and its start site was enriched with PolII, H3K4me3 and H3Ac, only one of the three intronic sites showed CTCF-enrichment.

Recent studies have reported genome wide surveys of both predicted [5] and experimentally verified [51] CTCF binding sites with 70 of them (45 experimentally verified, 25 predictions) in our region. Of the 45 experimentally identified CTCF binding sites, 32 were detected in our study in both cell lines (Figure S9). Lowering our detection threshold, it was possible to detect another 8 without including any false positives. No enrichment was seen on five of the reported in our study. When we looked at the 25 predicted CTCF binding sites, eight were detected in our study in both cells and a further, three in only one cell line. We detected an additional seven sites when we lowered the detection threshold (no extra false positives). Nine predicted sites did not show any enrichment in our study and one was not represented on our array.

A 2.2 kb long region (R7 in Table S5) was enriched with CTCF in both cell lines and it is a potential insulator since it lies between the intronic enhancer of the house-keeping gene *ADA* and tissue-specific promoter of *WISP2* (Figure 7). The intronic *ADA* enhancer is a strong regulatory element that can increase *ADA* promoter activity in a tissue-specific manner [45]. Enhancer elements exert their effects over long distances in an orientation-independent manner. So this enhancer can be a problem if it was to act on a promoter such as that of the *WISP2* which has a tissue-specific expression and is probably functioning in bone turnover [52]. An insulator element, as the one proposed here, can solve this problem by blocking the communication between the enhancer and the promoter located on the other side of the insulator.

There are five candidate insulator elements between 43.1 and 43.5 Mb (Figure S10). Each putative insulator lies between two candidate enhancers thereby possibly blocking the communication between these elements to ensure proper regulation of the neighbouring genes. It is important to note that all the genes in this window exhibit a tissues-specific expression pattern [53].

There are 18 CTCF-enriched regions located in introns (Table S6). *HNF4A*, *RIMS4*, *KCNS1* and *EYA2* are not expressed in either cell lines whereas *PKIG*, *STK4* and *DNTTIP* are expressed in both. Except *STK4*, none of the genes with an intronic CTCF-enrichment showed PolII or H3K4me3 enrichment on their start sites. These regions might be silencers as CTCF plays a major role as a repressor in silencer complexes [32], [50], [54].

TSSs of the three non-expressed genes (*KCNS1*, *WFDC10A* and *C20ORF165*) enriched with H3K4me3, but not with H3Ac, showed enrichments with CTCF in NT2/D1 cells (Table S6). The CTCF might play a role in these regions by inhibiting histone acetylation complexes. In HeLa S3, *KCNS1* and *WFDC10A* were also enriched with CTCF but did not show enrichment with H3K4me3 or H3Ac. This suggests that if CTCF functions as a repressor on the promoter of these genes its recruitment is probably independent of the region's H3K4me3 status since we detected binding in two different chromatin environments.

## Discussion

In this study, we present a partial histone map of a 3.5 Mb region of human chromosome 20q13.12 and identify novel promoter and distal regulatory elements. Analysis of histone modification profiles at TSSs verified that H3K4me1 does not have a discriminatory power for actively transcribing TSS, whereas tri-methylated H3K4 clearly marks active promoters. We found two novel tissue-specific promoters for *PKRCBP1* and *SULF2* by utilising the histone code of active promoters.

We went on to locate several putative tissue specific distal regulatory elements (enhancers, silencers and insulators) and we observed striking differences in the histone code profiles in the two cell lines examined (Figure 1 and 5). The differences in H3K27me3 profiles between the two cell lines suggested that H3K27me3 behaves as an antagonist of activatory histone modifications such as H3Ac, H3K4me2 and H3K4me3 in HeLa S3 cells whereas in NT2/D2 no such relation is observed (Figure 3 and Figure S4).

The 20q13.12 region has been implicated in several disease studies and the histone code, expression and reporter gene assays provided here would provide a framework to integrate sequence variation data and aid the design of functional studies to characterise disease causing variants. This systematic approach can be applied to all genomic intervals resulting from genome wide association studies of common disease and other complex traits.

## Supporting Information

Figure S1 Size distributions of ChIP samples prepared by crosslinking with 0.37% formaldehyde for 15 minutes from HeLa S3 (Lane 11) and NT2/D1 (Lane 12) cells run on Agilent BioAnalyser.
(0.04 MB TIF)

Figure S2 Histograms of enrichment signals of all antibodies in HeLa S3 and NT2/D1 cells. The overlapping bars are shown in dark green.
(1.41 MB TIF)

Figure S3 Pairwise correlation coefficient matrix of enrichment signals for all antibodies on the spots containing TSSs in HeLa S3 cells (green rectangle), NT2/D1 cells (red rectangle), and between the two cell lines (remaining areas).
(0.89 MB TIF)

Figure S4 Pairwise correlation coefficient matrix of enrichment signals for all antibodies on all the spots in HeLa S3 cells (green rectangle), NT2/D1 cells (red rectangle), and between the two cell lines (remaining areas). Note that only coefficients which are statistically significant at the 95% level, according to a standard p-test are shown as non-zero.
(0.90 MB TIF)

Figure S5 Upstream region of PRKCBP1 (chr20:45,431,095–45,431,820 bp) was cloned to pGL3-basic vectors in both directions (S;sense, AS:antisense) and transfected to both cells together with internal control plasmid. The promoter construct in the antisense direction (in the same direction with PRKCBP1) showed ∼18 fold promoter activity compared to null and negative control constructs in NT2/D1 cells but no significant activity was observed in HeLa S3 cells.
(0.16 MB TIF)

Figure S6 Promoter assays of a region within the first intron of SULF2 (chr20:45,818,232–45,819,183 bp) in both cell lines. The region was cloned to pGL3-basic vectors in both directions (S;sense, AS:antisense) and transfected to both cells together with internal control plasmid. The construct in the antisense direction (in the same direction with SULF2) showed ∼2-fold promoter activity compared to null construct in NT2/D1 cells.
(0.12 MB TIF)

Figure S7 Dual Luciferase Assays of a segment of the region spanning chr20:42,749,148–42,766,245 bp (denoted as put_enhancer). It showed around 5 fold activity than randomly chosen inter-genomic fragments (of same length) in both cell lines.
(0.10 MB TIF)

Figure S8 Enrichment profile of SLC12A5 with H3Ac, H3K4me2, H3K4me3 and H3K27me3 antibodies in both cell lines.
(1.00 MB TIF)

Figure S9 CTCF binding sites reported in our study and CTCFBSDB.
(2.58 MB TIF)

Figure S10 The region spanning from 43,100,000 to 43,425,000 bp where there are five candidate insulators shown as blue boxes on the insulator track. There are two more tracks, displaying H3K4me1 and H3K4me2 enriched regions in HeLa S3 (Enhancers_H track) and NT2/D1 (Enhancers_N track) as possible cis-acting regulatory elements.
(0.43 MB TIF)

Table S1 Enrichment profile of 70 transcripts in HeLa S3 cells. “*” denotes enrichment above threshold.
(0.02 MB XLS)

Table S2 Enrichment profile of 70 transcripts in NT2/D1 cells. “*” denotes enrichment above threshold.
(0.04 MB XLS)

Table S3 Genes sorted according to their expression levels in both cells (X-axis of Figure 1)
(0.04 MB XLS)

Table S4 Enrichment profile of H3K27me3-enriched regions in HeLa S3 cells. “*” denotes enrichment above threshold.
(0.02 MB XLS)

Table S5 Enrichment profile of H3K27me3-enriched regions in NT2/D1 cells. “*” denotes enrichment above threshold.
(0.04 MB XLS)

Table S6 List of regions and their enrichment profiles enriched with CTCF in HeLa S3 and NT2/D1 cells. “*” denotes enrichment above threshold.
(0.05 MB XLS)
