# Supplementary figures and images for: A Histone Map of Human Chromosome 20q13.12
Source: PLoS One. 2009 Feb 20;4(2):e4479. doi: 10.1371/journal.pone.0004479 (PMC2639704; doi:10.1371/journal.pone.0004479)

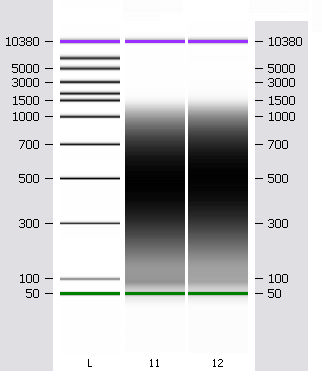

Supplement: Figure S1 — Size distributions of ChIP samples prepared by crosslinking with 0.37% formaldehyde for 15 minutes from HeLa S3 (Lane 11) and NT2/D1 (Lane 12) cells run on Agilent BioAnalyser. (0.04 MB TIF) [file pone.0004479.s001.tif]

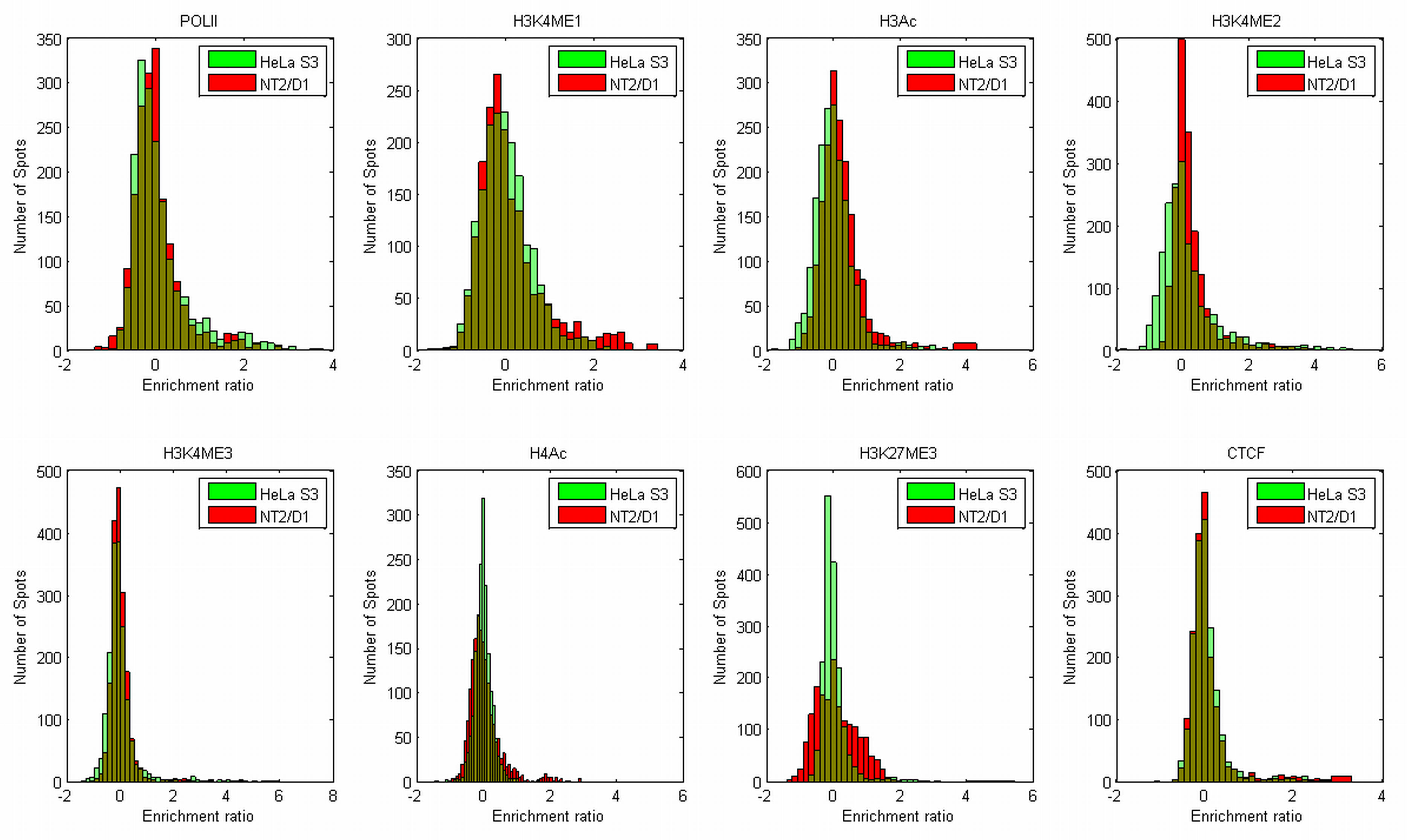

Supplement: Figure S2 — Histograms of enrichment signals of all antibodies in HeLa S3 and NT2/D1 cells. The overlapping bars are shown in dark green. (1.41 MB TIF) [file pone.0004479.s002.tif]

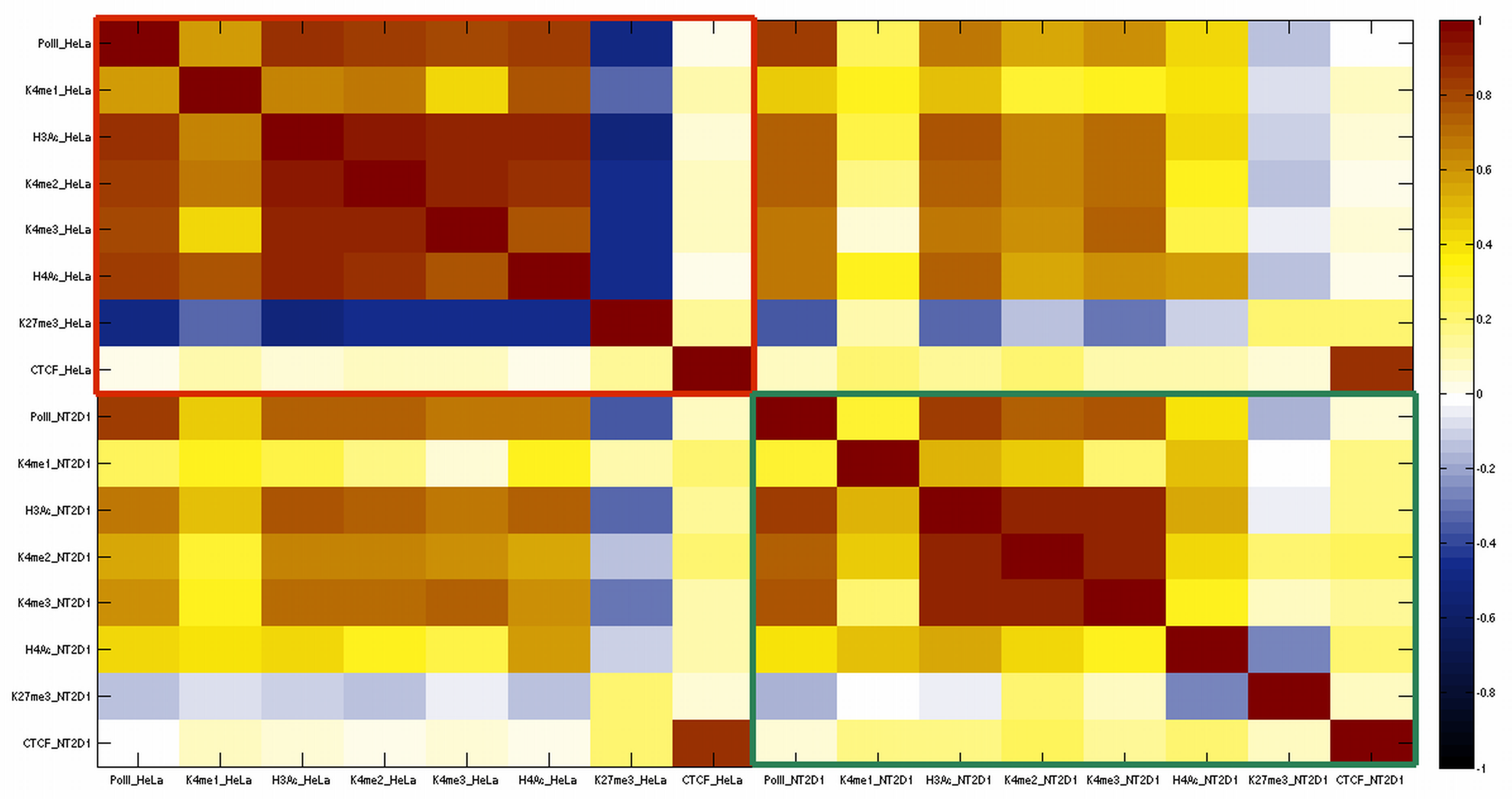

Supplement: Figure S3 — Pairwise correlation coefficient matrix of enrichment signals for all antibodies on the spots containing TSSs in HeLa S3 cells (green rectangle), NT2/D1 cells (red rectangle), and between the two cell lines (remaining areas). (0.89 MB TIF) [file pone.0004479.s003.tif]

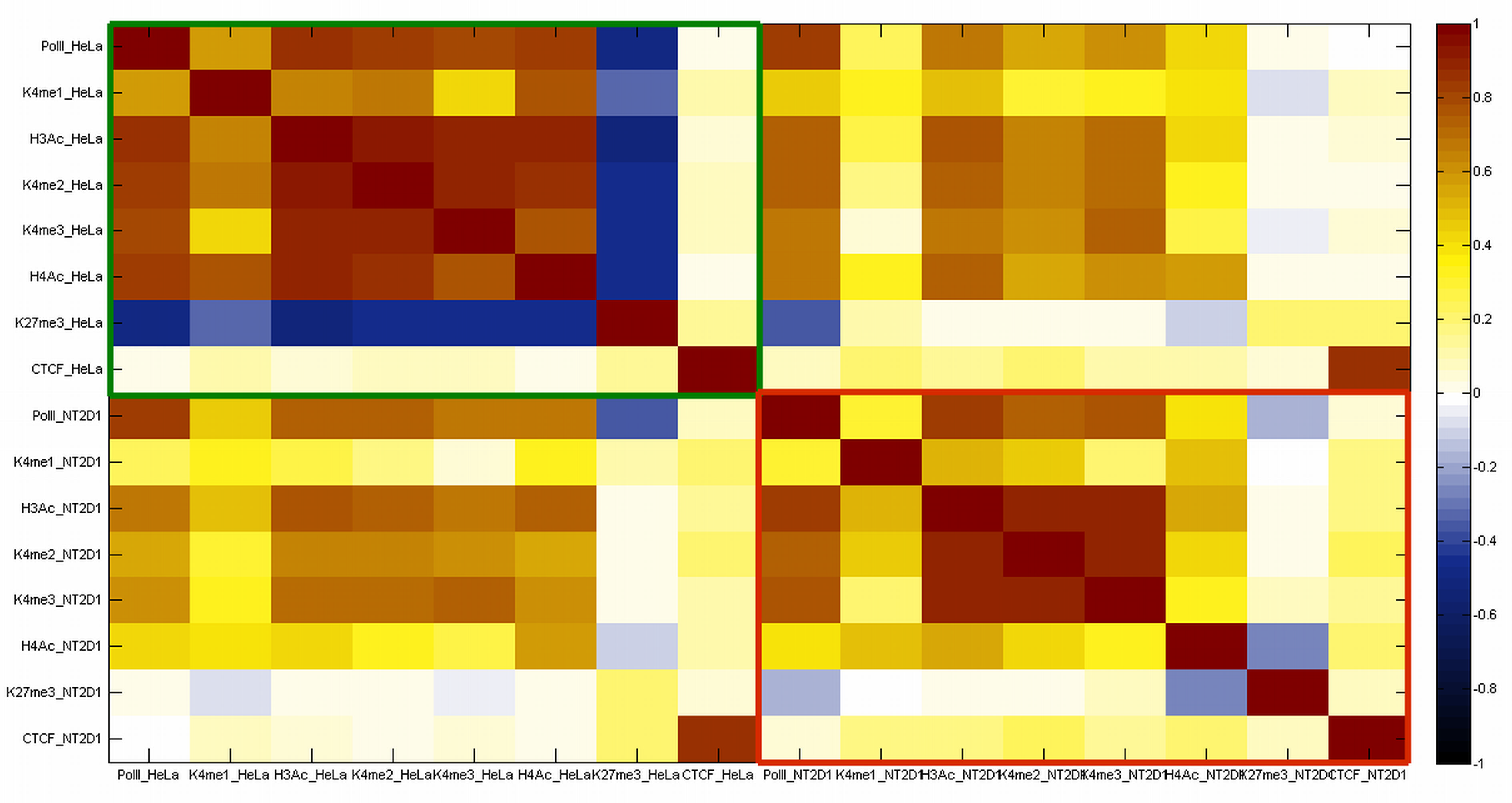

Supplement: Figure S4 — Pairwise correlation coefficient matrix of enrichment signals for all antibodies on all the spots in HeLa S3 cells (green rectangle), NT2/D1 cells (red rectangle), and between the two cell lines (remaining areas). Note that only coefficients which are statistically significant at the 95% level, according to a standard p-test are shown as non-zero. (0.90 MB TIF) [file pone.0004479.s004.tif]

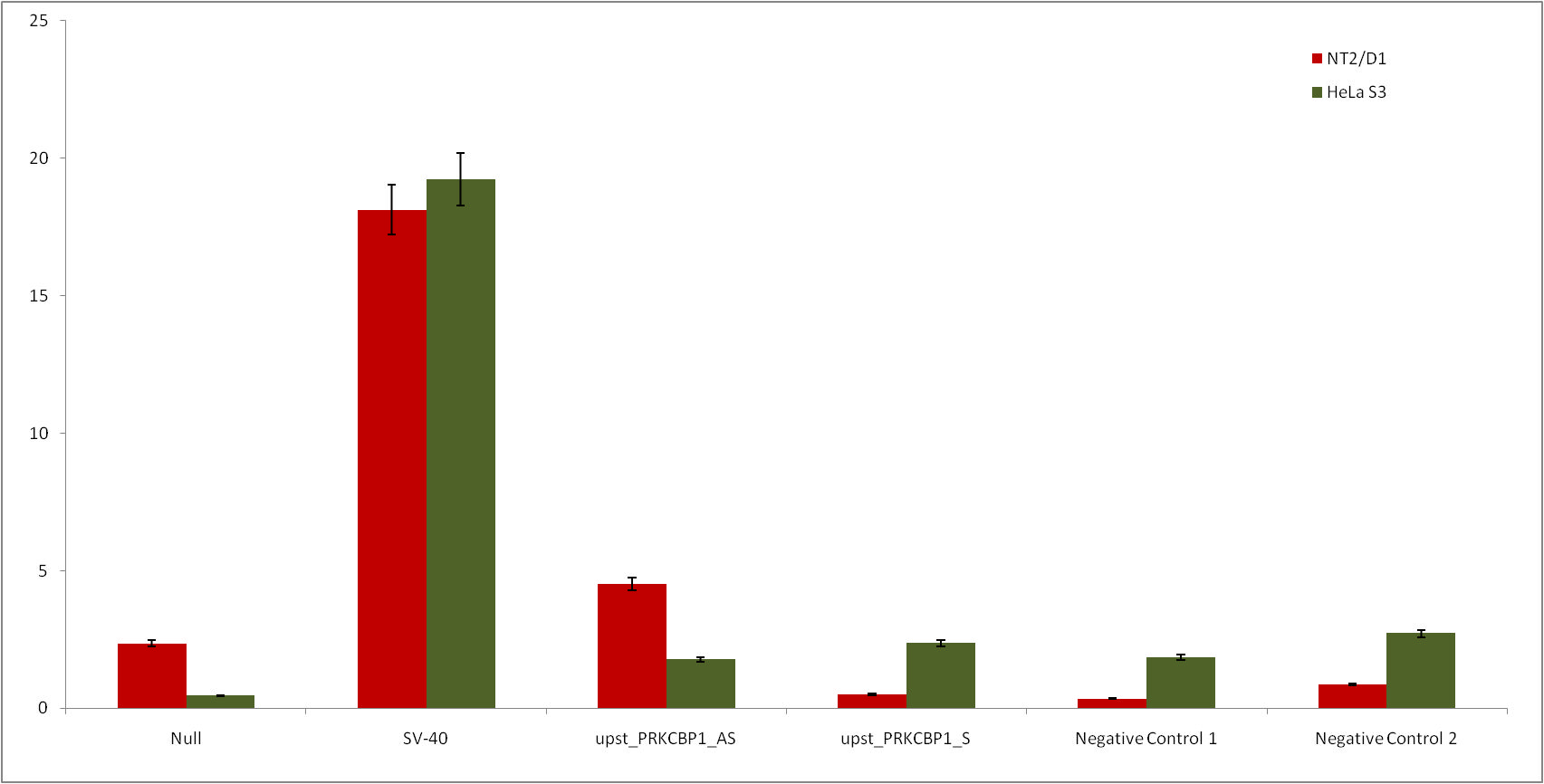

Supplement: Figure S5 — Upstream region of PRKCBP1 (chr20:45,431,095–45,431,820 bp) was cloned to pGL3-basic vectors in both directions (S;sense, AS:antisense) and transfected to both cells together with internal control plasmid. The promoter construct in the antisense direction (in the same direction with PRKCBP1) showed ∼18 fold promoter activity compared to null and negative control constructs in NT2/D1 cells but no significant activity was observed in HeLa S3 cells. (0.16 MB TIF) [file pone.0004479.s005.tif]

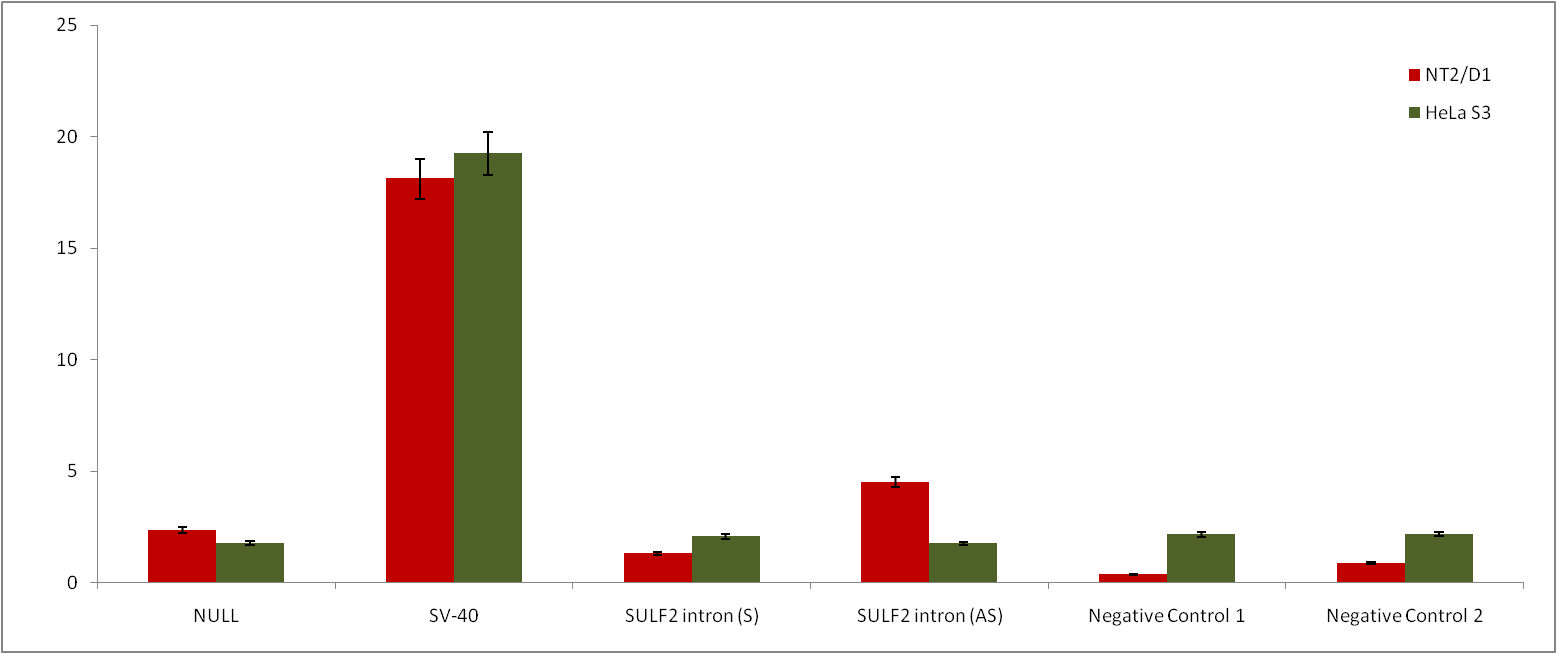

Supplement: Figure S6 — Promoter assays of a region within the first intron of SULF2 (chr20:45,818,232–45,819,183 bp) in both cell lines. The region was cloned to pGL3-basic vectors in both directions (S;sense, AS:antisense) and transfected to both cells together with internal control plasmid. The construct in the antisense direction (in the same direction with SULF2) showed ∼2-fold promoter activity compared to null construct in NT2/D1 cells. (0.12 MB TIF) [file pone.0004479.s006.tif]

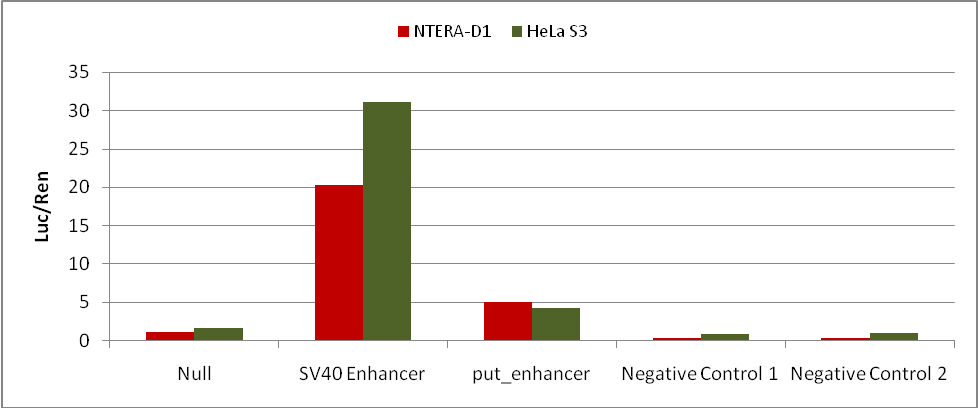

Supplement: Figure S7 — Dual Luciferase Assays of a segment of the region spanning chr20:42,749,148–42,766,245 bp (denoted as put_enhancer). It showed around 5 fold activity than randomly chosen inter-genomic fragments (of same length) in both cell lines. (0.10 MB TIF) [file pone.0004479.s007.tif]

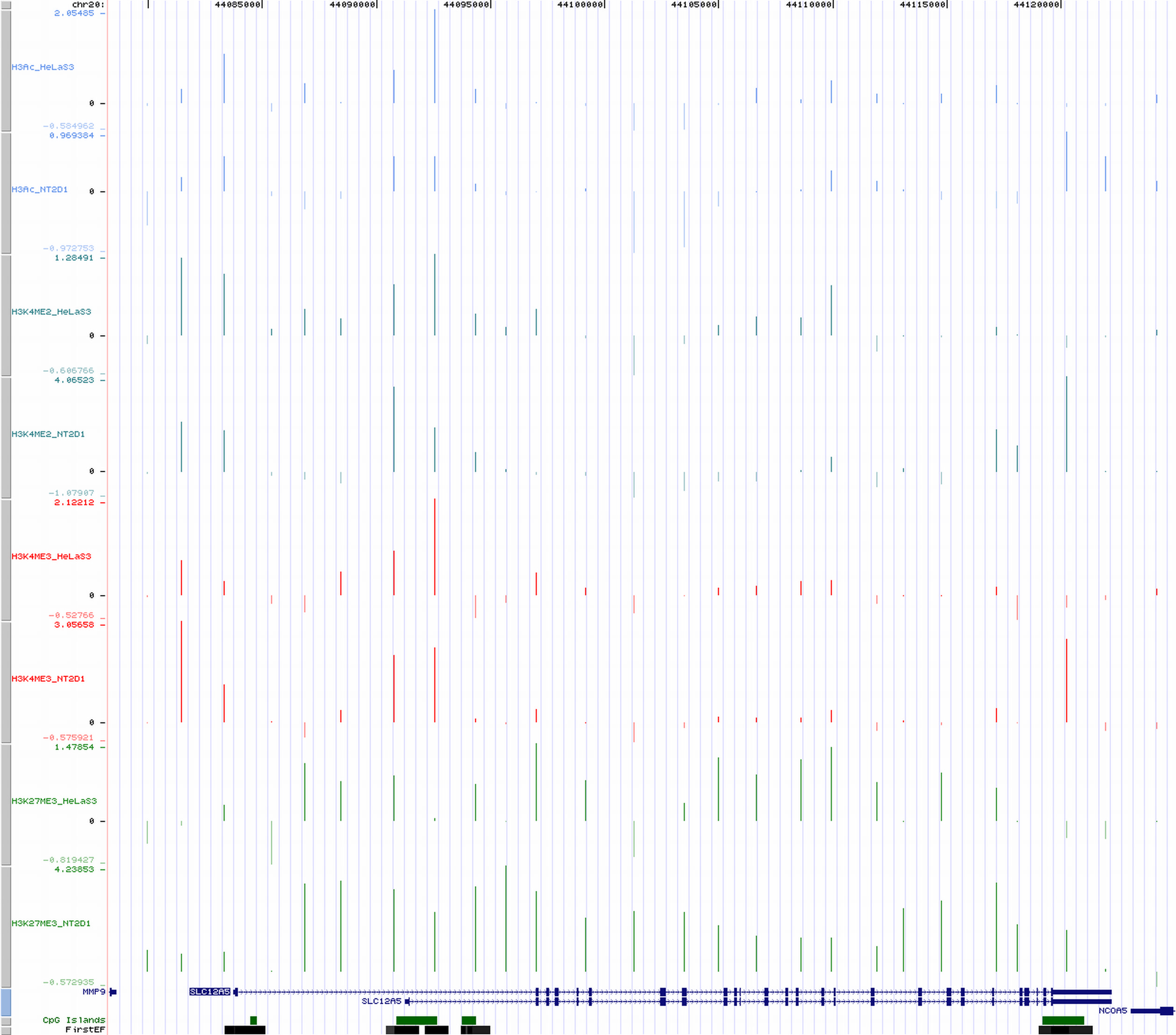

Supplement: Figure S8 — Enrichment profile of SLC12A5 with H3Ac, H3K4me2, H3K4me3 and H3K27me3 antibodies in both cell lines. (1.00 MB TIF) [file pone.0004479.s008.tif]

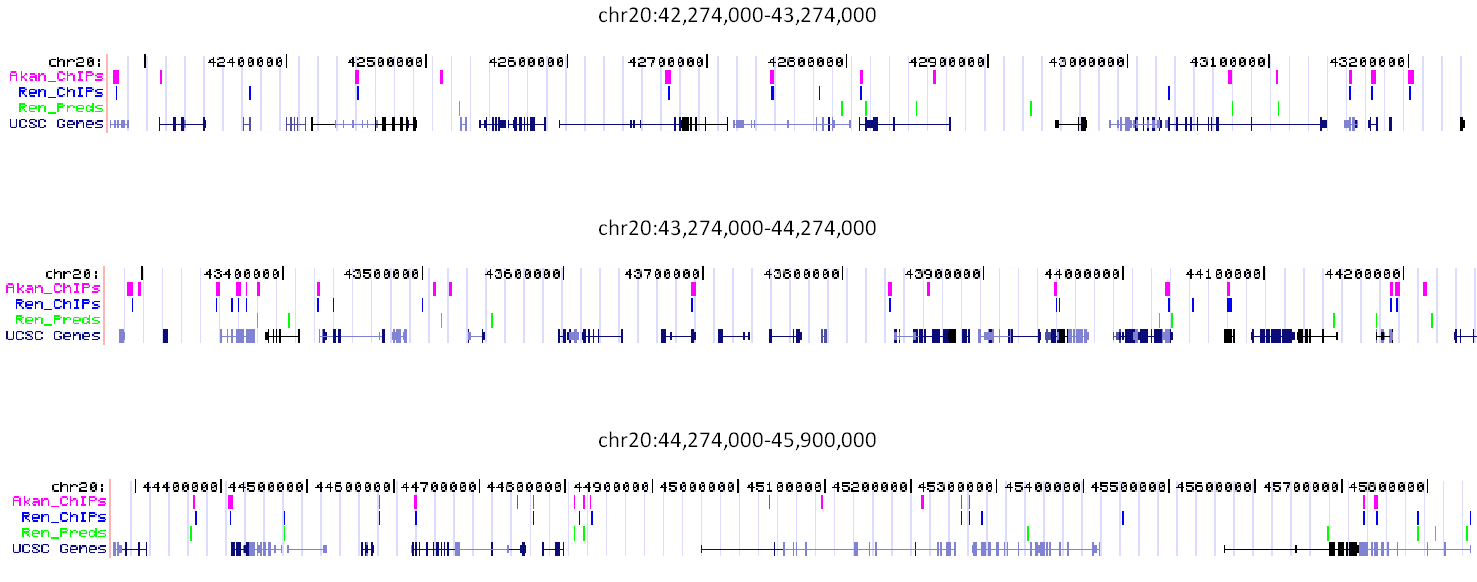

Supplement: Figure S9 — CTCF binding sites reported in our study and CTCFBSDB. (2.58 MB TIF) [file pone.0004479.s009.tif]

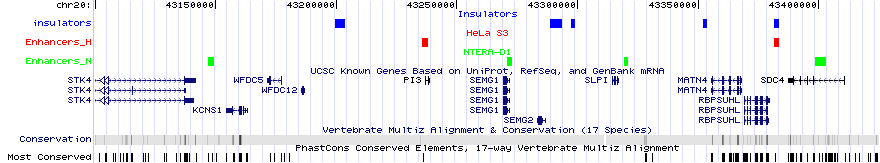

Supplement: Figure S10 — The region spanning from 43,100,000 to 43,425,000 bp where there are five candidate insulators shown as blue boxes on the insulator track. There are two more tracks, displaying H3K4me1 and H3K4me2 enriched regions in HeLa S3 (Enhancers_H track) and NT2/D1 (Enhancers_N track) as possible cis-acting regulatory elements. (0.43 MB TIF) [file pone.0004479.s010.tif]
